# An empirical study for determining the quality indicators for the primary and secondary school of Bangladesh: A structural equation modeling approach

**DOI:** 10.1016/j.heliyon.2022.e10870

**Published:** 2022-10-03

**Authors:** Suman Ahmmed, Jashodhan Saha, Maruf Ahmed Tamal

**Affiliations:** a Department of Computer Science & Engineering, United International University, Dhaka, Bangladesh; b Institute of Natural Sciences, United International University, Dhaka, Bangladesh; c Institute for Advanced Research, United International University, Dhaka, Bangladesh

**Keywords:** Student achievement, Teacher quality, School environment, Technology, Leadership, PLS-SEM, School quality, Bangladesh

## Abstract

Primary and secondary school quality indicators need to be defined, assessed, and monitored due to their incomparable contributions to the entire educational process as well as for the overall development of a country. However, previous studies rarely emphasized indicators that have a substantial impact on school quality, especially in the context of developing countries. Bangladesh is one of them where this has yet to be adequately studied. To bridge this gap, this study aimed to explore the key “driver” constructs of primary and secondary level school quality. Adopting a quantitative cross-sectional research design, the present study was conducted between April 2021 to January 2022 at 128 primary and secondary level schools in 38 different districts of Bangladesh. Data were analyzed using Partial Least Squares Structural Equation Modeling (PLS-SEM). Empirical findings revealed that teacher quality (β = 0.588, t = 12.242, p < 0.001), technology (β = 0.279, t = 5.402, p < 0.001), school leadership (β = 0.132, t = 2.383, p < 0.05) have positive influence on students' academic achievement which ultimately affects (β = 0.923, t = 92.713, p < 0.001) the overall quality of the school. While school environment does not significantly influence students’ academic achievement or school quality. The overall findings of this study are expected to assist the devolved authorities in implementing synchronized policies for the improvement of primary and secondary level school quality.

## 1 Introduction

Education is a dynamic process of assisting humans in gaining new skills, knowledge, values, beliefs, and habits [1]. Quality education is the precondition of any human resource development, and that is why every human being is entitled to this basic human right. The International Standard Classification of Education (ISCED) classifies formal educational attainment into nine levels, the first of which is early childhood education and then continues up to a doctoral degree [2]. Among all, the primary and secondary levels are deemed to be the fundamental levels since these two directly affect the country’s sustainable economic development and poverty reduction [3, 4, 5, 6]. That’s why it’s so important to ensure the school’s quality to enhance the success probability of the students, particularly at the primary and secondary levels. However, defining school quality is perplexing and often difficult to measure due to its complex nature and quantifying complexity. Consequently, when addressing what constitutes a quality school, the indicators of quality defined by policymakers, parents, and community members often differ, and no one ever comes to the same conclusion [7]. As an example, when it comes to evaluating a school’s quality, policymakers frequently depend on students’ test scores and their grades, whereas community members, parents, and other stakeholders prefer to rely on a school’s facilities, school’s reputation, word of mouth, and their observations [8].

To define why some schools may be better than others, the report of the National Center for Education Statistics (NCES) [9] identified teachers as one of the key indicators of school quality. The report showed that teachers with strong academic skills, prior teaching experience, and training have a significant impact on school quality through effective teaching. Also, the report stated that school quality is inseparable from teacher quality because it directly and indirectly affects student learning. Similar findings were obtained in a study [10], where the authors argued that teacher quality has a significant and meaningful impact on students’ learning outcomes. The study found that students’ learning processes are significantly improved by teachers with teaching experience compared to those with no prior experience. This may be because teachers’ productivity and teaching effectiveness increase with experience, as evident in the previous studies [11, 12].

Prior research studies have also identified technology as a significant indicator in determining a school’s quality, as the usage of technology enhances students’ learning engagement [9, 13, 14, 15]. The use of technology in education has been more prominent than ever before in recent years. Consequently, there is a growing amount of research on the use of digital technology in schools, classrooms, among teachers and students. Currently, to enhance the capabilities of the next generation and better pedagogical and academic achievement, nations (such as Iran, Malaysia & many developing countries) throughout the world are embracing the Technology-based Smart School (TSS) idea [16]. As stated in the study [16], Smart School-an appropriate multimedia software and training for teachers, as well as educational technology, boosts students’ learning and retention in areas like mathematics. In addition, technology-enhanced education improves teacher-student communication, enhances students’ learning autonomy, and provides innovative thinking for accomplishing students’ deep learning [17].

Leadership is another well-recognized quality indicator for schools worldwide [18]. This role is challenging because it encompasses strategy, culture, relationships, administration, complicated decision-making, and often conflicting stakeholder perspectives in school [19]. However, despite being complex in nature, every school needs a competent individual or group of individuals who can give direction, guidance, and support along school’s path toward its goals [9]. Several studies [20, 21, 22, 23, 24, 25, 26] have been conducted to investigating the impact of school leadership (both instructional and transformational) on student achievement in different countries. Among them, the majority of the studies [21, 25, 26] identified Instructional Leadership (IL) effects on student achievement more compared to Transformational Leadership (TL). IL is primarily concerned with enhancing the quality of teaching and learning by defining the goals of the school, overseeing the program, and fostering a supportive learning environment. In contrast, TL was to have leaders who strategically improve a school by boosting the achievement and motivation of their teachers. However, few studies [23, 24] found that TL had a greater impact on students' achievement than IL.

Despite being complex and controversial [27], previous studies suggested that the school environment (both indoor-classroom size, lighting, color, infrastructure, ventilation, safety, etc. and outdoor-green schoolyard, garden, spacious campus, playground, etc.) has a significant and crucial effect on students’ academic performance. For example, by referring to the school as a student’s second home, the study [28] asserted that the school environment has a significant impact on students’ learning experiences and, subsequently, academic outcomes. In another study [29], the authors observed that a positive school atmosphere not only enhances engagement in school activities but also assures students’ mental well-being, which eventually impacts their academic performance.

In the educational process, it’s evident to everyone, from policymakers to parents, that schools have a significant role in the success of their children. Throughout the years, numerous studies have been undertaken to find indicators that might predict a student’s academic achievement but few have attempted to find indicators that play a significant influence in determining a school’s quality. At the same time, as far as researchers’ knowledge, the majority of prior studies have focused on western countries. In contrast, developing countries like Bangladesh are far behind. So, for expanding educational innovations, this study determined that it was necessary to explore what aspects play an important role in defining school quality in the context of Bangladesh, especially at the primary and secondary levels.

The remaining sections of the study are organized as follows: In Section 2, the materials and methods used in this study are briefly discussed. The results of this study are presented step-by-step in Section 3. Section 4 offers an interpretation of the results in light of previous studies. In Section 5, based on the findings of the present study, recommendations and policy implications are suggested. The executive summary of the study is emphasized in Section 6. In Section 7, the limitations of the study and potential directions for future research are discussed, and finally, the authors thank the supporting organizations for their contributions to the study in Section 8.

## 2 Materials and methods

### 2.1 Conceptual model and hypotheses

**Figure 1:**
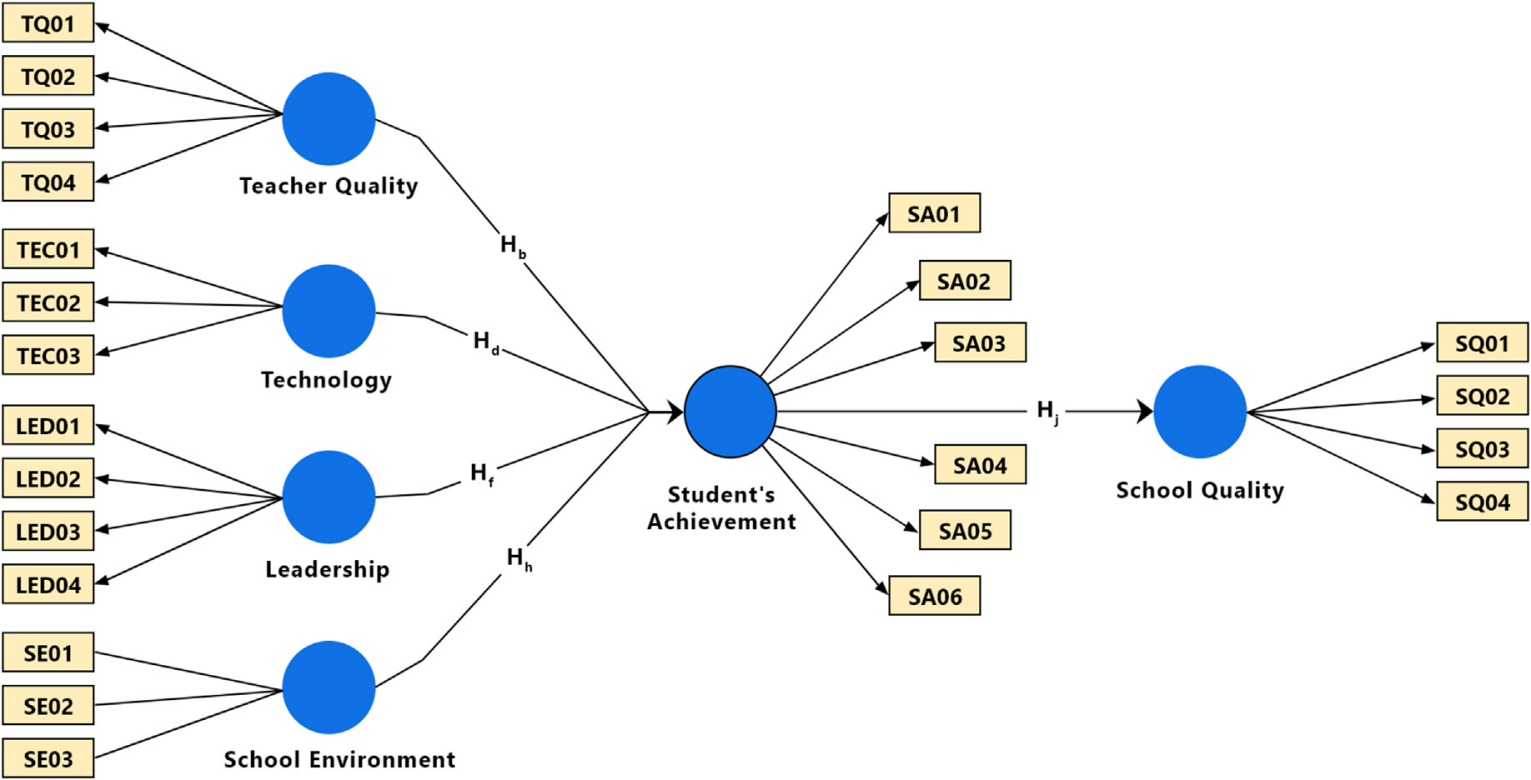
Conceptual framework.

To explore the predicting indicators of primary and secondary level school quality, a conceptual model is proposed and tested in this study (see Figure 1). It contains six constructs: (i) teacher quality, (ii) technology, (iii) leadership, (iv) school environment, (v) student’s achievement and (vi) school quality. Among the constructs, 4 (i–iv) are exogenous (independent) and 2 (v and vi) are endogenous (dependent). However, the construct of student’s achievement (v) has dual relationships as both independent and dependent. This is because this construct is predicted by 4 (i–iv) exogenous constructs, while it also predicts another endogenous construct (vi) simultaneously. Decisions concerning the inclusion of constructs and directional relationships within the model were guided by theoretical considerations from the literature review [9, 10, 11, 12, 13, 14, 15, 16, 17, 18, 19, 20, 21, 22, 23, 24, 25, 26, 27, 28, 29]. Finally, according to the conceptual model illustrated in Figure 1, the study hypotheses can be formed as follows:

$$H_{a}=\;Teacher\;quality\;does\;not\;impact\;on\;students’\;achievement.$$

$$H_{b}=\;Teacher\;quality\;does\;impact\;students’\;achievement.$$

$$H_{c}=\;Technology\;\left(in\;school\right)\;does\;not\;impact\;on\;students’\;achievement$$

$$H_{d}=\;Technology\;\left(in\;school\right)\;does\;impact\;students’\;achievement$$

$$H_{e}=\;School\;leadership\;does\;not\;impact\;on\;students’\;achievement.$$

$$H_{f}=\;School\;leadership\;does\;impact\;students’\;achievement.$$

$$H_{g}=\;School\;environment\;does\;not\;impact\;on\;students’\;achievement.$$

$$H_{h}=\;School\;environment\;does\;impact\;students’\;achievement.$$

$$H_{i}=\;Student’s\;achievement\;does\;not\;impact\;on\;school\;quality.$$

$$H_{j}=\;Student’s\;achievement\;does\;impact\;school\;quality.$$

### 2.2 Study design and settings

Adopting a quantitative cross-sectional research design, this study was conducted between April 2021 and January 2022 in 128 primary and secondary level schools in 38 different districts of Bangladesh.

### 2.3 Population and sample size

**Figure 2:**
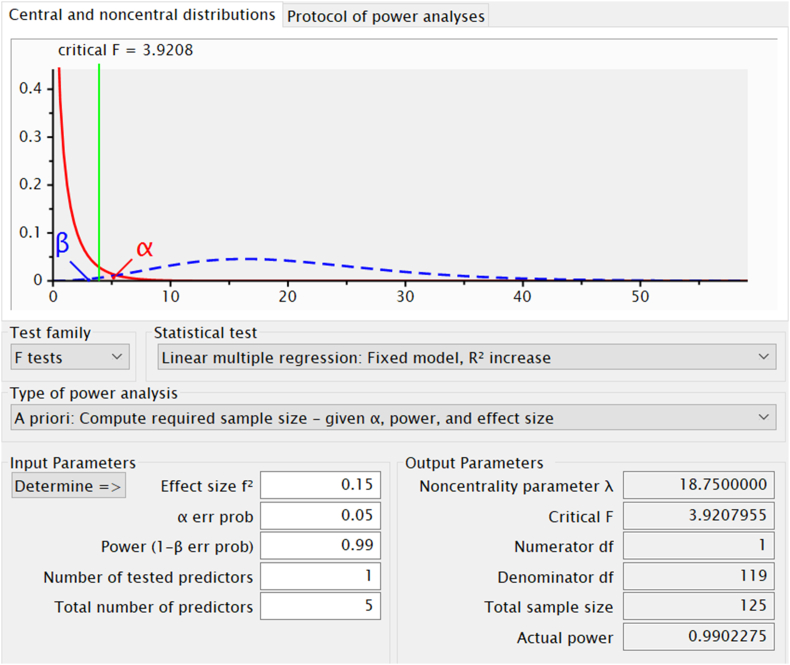
Power result of required sample size.

The target population of this study is the primary and secondary level schools in Bangladesh, totaling 65566 and 20849, respectively, until 2020 [30]. However, the sample size of this study comprised 128 randomly selected schools, where 21 (16.4%) were at the primary level, while 107 (83.6%) were at the secondary level. 38.3% of schools were from rural areas, while 51.6 % and 10.2% of schools were from suburban and urban areas, respectively. Though the sample size may appear small to infer the population, this study adopted Partial Least Squares Structural Equation Modeling (PLS-SEM), a robust multivariate analysis technique, well recognized for its high levels of statistical power despite having a small sample [31, 32]. To estimate sample size in PLS-SEM, scholars use several benchmarks. Among them, the often-cited 10 times rule is a widely used minimum sample size estimation method in which the sample size has to be at least ten times the indicators used to measure the construct [33]. However, the studies [34] and [35] claimed the 10 times rule can lead to grossly inaccurate estimations of the minimum required sample size. Hence, this study employed G ∗ Power (version 3.1.9.7) [36], an often used [37, 38] and recommended tool [32] to estimate the minimum sample size. As the proposed conceptual model of this study has 5 constructs (predictors of school quality), the authors selected the effect size (f2) as medium 0.15, α err prob as 0.05, and the power (1-β err prob) as 0.99 (see Figure 2) recommended by the previous studies [32, 33, 36, 37]. Finally, the calculation ended up with the required sample size of 125. Hence, the number of samples (n = 128) in this study is adequate to ensure sufficient statistical power.

### 2.4 Data collection instrument and procedure

The primary data for this study was collected between April 2021 and January 2022 from 128 primary and secondary schools in 38 different districts of Bangladesh. The questionnaire, which is provided in Appendix A, was designed and developed by quantitative research experts based on the drivers of school quality (e.g., teacher quality, school environment, technology, leadership, and student’s achievement). It was consisted of 28 close-ended questions, which were split into 2 major sections. The first section was intended to collect schools' basic information such as school’s name, location, level, etc. While the second section had 23 questions related to the drivers of school quality. Majority of the questions were based on 5-point rating scale ranging from strongly disagree to strongly agree. While a few were based on a 10-point scale. The validity and reliability of the questionnaire were confirmed by content validity and internal consistency reliability, respectively. Content validity was ensured by the judgment method. Following the recommendation of the study [39], a panel of 6 experts (practitioners from the education field) used a 5-point rating scale to evaluate the questionnaire on dimensions such as relevance, representativeness, specificity, and clarity. To quantify the expert agreement, Cohen’s coefficient kappa (k) was used as suggested in a study [40]. The kappa (k) value of the questionnaire was .87 (significance<.05∗∗), showing a substantial level of agreement [41]. Besides, to estimate the items-internal consistency reliability, Cronbach’s Alpha coefficient is considered [42]. Findings revealed that Cronbach’s Alpha coefficient of the questionnaire was .803, indicating acceptable internal consistency reliability [43]. To collect the data, a team of expert researchers had to go to each of the schools and collect the required information. The necessary approval was obtained from corresponding institutional authorities: Institute for Advanced Research (IAR), United International University (Reference: UIU/IAR/01/2021/SE/03) as well as consent was obtained from the heads of all participating schools. Data was initially gathered using a handy paper and pencil approach and later input into Excel for further analysis.

### 2.5 Data analysis

The data was analyzed in two stages. Initially, a suitable descriptive analysis was performed using IBM SPSS Statistics 26. Later, PLS-SEM [32], a sophisticated second-generation multivariate analysis method was employed using SmartPLS (version: 3.3.9) [44] for exploring the key “driver” constructs of school quality. PLS-SEM is a variance-based method to estimate structural equation models. It consists of two models, firstly, the measurement model (also known as the outer model) and secondly the structural model (also called the inner model). PLS-SEM is primarily used to analyze complex inter-relationships between observed and latent variables. To develop theories in exploratory research and simultaneously analyze the complex relationships among multiple variables [32], researchers frequently use this technique. In this study, the choice of PLS-SEM is justified since it provides more flexibility to explore and experiment with numerous configurations [45], especially when the sample size is small [32]. In addition, referring to [46], PLS-SEM is more suitable compared to other methods (e.g., CB-SEM) when the goal is to predict the key “driver” constructs. In this study, the major parameter settings of SmartPLS are as follows: (i) Algorithm to handle missing data: None; Data metric: Mean 0, Var 1; Initial weights: 1; Max. number of iterations: 300; Stop Criterion (10ˆ-X): 7; Use Lohmoeller settings? No, and Weighting scheme: Path.

## 3 Results

### 3.1 Evaluation of measurement model

The first step in evaluating PLS-SEM results involves examining the measurement models [47]. According to [32, 48], and [49], the standard assessment criteria, which should be considered to assess the reflective measurement model includes three aspects, namely internal consistency reliability, convergent validity, and discriminant validity.

#### 3.1.1 Internal consistency reliability

Internal consistency refers to the degree of homogeneity between the observed indicator variables in a test or questionnaire. The traditional criterion for determining internal consistency reliability is Cronbach’s alpha and Composite reliability. However, as compared to Cronbach’s alpha, composite reliability is more appropriate since it prioritizes the indicators based on their individual reliability [32]. The value of composite reliability varies from 0 to 1, where higher values indicate a higher degree of reliability. Therefore, in exploratory research, composite reliability >.60 is acceptable [32, 37]. Table 1 demonstrates that all of the constructs exceeded the recommended threshold level (>.60). Thus, the sample’s required internal consistency reliability is ensured.

**Table 1:** Validity and reliability of measurement model.

Constructs	Indicators	Convergent validity					Internal consistency reliability	
		Std. Dev.	Mean	Loading	AVE	P Value	Cronbach’s Alpha	Composite Reliability
Technology	TEC01	0.022	0.878	0.877	0.71	0.000	0.792	0.879
	TEC02	0.051	0.742	0.744		0.000		
	TEC03	0.013	0.899	0.899		0.000		
Teacher Quality	TQ01	0.015	0.895	0.895	0.799	0.000	0.916	0.941
	TQ02	0.018	0.901	0.903		0.000		
	TQ03	0.016	0.899	0.900		0.000		
	TQ04	0.022	0.877	0.878		0.000		
Leadership	LED01	0.017	0.917	0.917	0.774	0.000	0.900	0.931
	LED02	0.055	0.722	0.726		0.000		
	LED03	0.01	0.945	0.946		0.000		
	LED04	0.012	0.913	0.913		0.000		
School Environment	SE01	0.009	0.95	0.95	0.862	0.000	0.92	0.949
	SE02	0.013	0.929	0.931		0.000		
	SE03	0.014	0.904	0.904		0.000		
Student’s Achievement	SA01	0.025	0.763	0.762	0.708	0.000	0.915	0.935
	SA02	0.037	0.782	0.785		0.000		
	SA03	0.008	0.94	0.94		0.000		
	SA04	0.02	0.886	0.888		0.000		
	SA05	0.046	0.686	0.689		0.000		
	SA06	0.006	0.952	0.951		0.000		
School Quality	SQ01	0.008	0.948	0.948	0.864	0.000	0.947	0.962
	SQ02	0.015	0.911	0.912		0.000		
	SQ03	0.015	0.915	0.916		0.000		
	SQ04	0.009	0.94	0.941		0.000		

#### 3.1.2 Convergent validity

Convergent validity refers to how well a measure correlates with alternative measures of the same construct to explain the variance of its measures. It implies that measures with similar or identical constructs should be substantially related [38]. The outer loadings of the indicators, as well as the average variance extracted (AVE), are widely utilized by scholars to determine convergent validity [32, 48]. The suggested rule of thumb is that the outer loadings and AVE values should be at least 0. 708 and 0.50, respectively [32, 37], and [48]. Table 1 shows that the AVEs exceeded 0.5 for all constructs, meaning each construct explains over 50% of the variance of its items. Similarly, all the outer loadings also met the suggested threshold except SA05 (outer loading = 0.689). However, SA05 was not removed since it did not increase the AVE. Thus, establishing the convergent validity of the measurement model.

#### 3.1.3 Discriminant validity

Discriminant validity is the extent to which a construct is empirically distinct from other constructs [32, 48]. The main purpose of discriminant validity is to ensure whether a reflective construct has the strongest correlations with its indicators. Generally, scholars use two measures to assess discriminant validity: (i) by examining indicators' cross-loadings and (ii) by the Fornell-Larcker criterion [47]. Referring to [32], the indicator’s cross-loadings on the associated construct should be higher than all other indicators' loadings of other constructs. Otherwise, it’s a problem with discriminant validity. Table 2 presents the cross-loadings of all indicators that met the suggested threshold.

**Table 2:** Discriminant validity (cross-loading).

Items	Constructs					
	Leadership	School Environment	School Quality	Student’s Achievement	Teacher Quality	Technology
LED01	**0.917**	0.672	0.808	0.785	0.765	0.667
LED02	**0.726**	0.538	0.623	0.533	0.600	0.543
LED03	**0.946**	0.677	0.813	0.785	0.752	0.686
LED04	**0.913**	0.753	0.832	0.772	0.780	0.736
SA01	0.536	0.602	0.594	**0.762**	0.613	0.586
SA02	0.573	0.661	0.659	**0.785**	0.712	0.689
SA03	0.796	0.866	0.912	**0.940**	0.910	0.842
SA04	0.701	0.696	0.804	**0.888**	0.802	0.693
SA05	0.696	0.589	0.684	**0.689**	0.615	0.533
SA06	0.826	0.881	0.935	**0.951**	0.920	0.871
SE01	0.673	**0.950**	0.735	0.790	0.797	0.818
SE02	0.635	**0.931**	0.700	0.755	0.721	0.788
SE03	0.783	**0.904**	0.892	0.852	0.856	0.800
SQ01	0.83	0.802	**0.948**	0.846	0.866	0.748
SQ02	0.846	0.720	**0.912**	0.818	0.826	0.691
SQ03	0.761	0.757	**0.916**	0.822	0.830	0.771
SQ04	0.831	0.837	**0.941**	0.938	0.901	0.79
TEC01	0.707	0.795	0.743	0.731	0.721	**0.877**
TEC02	0.418	0.557	0.495	0.626	0.495	**0.744**
TEC03	0.744	0.811	0.779	0.778	0.781	**0.899**
TQ01	0.763	0.761	0.826	0.826	**0.895**	0.724
TQ02	0.755	0.773	0.835	0.816	**0.903**	0.709
TQ03	0.755	0.785	0.832	0.81	**0.900**	0.717
TQ04	0.686	0.743	0.804	0.837	**0.878**	0.711

Furthermore, the Fornell-Larcker criterion [47] is an often-cited and more conservative measure for assessing discriminant validity. It mainly compares the square root of AVE values with the construct variable correlations [32]. To achieve discriminant validity, the square root of each construct’s AVE should be greater than its highest correlation with any other construct within the same model [32, 47, 50]. Table 3 shows the same findings as recommended. Therefore, the discriminant validity of the measurement model was confirmed by both the cross-loading and the Fornell-Larcker criterion.

**Table 3:** Discriminant validity (Fornell-Larcker).

	LED	SE	SQ	SA	TQ	TEC
Leadership (LED)	0.880	_				
School Environment (SE)	0.755	0.928	_			
School Quality (SQ)	0.880	0.840	0.929	_		
Student’s Achievement (SA)	0.827	0.863	0.924	0.841	_	
Teacher Quality (TQ)	0.827	0.856	0.922	0.920	0.894	_
Technology (TEC)	0.751	0.865	0.808	0.848	0.80	0.843

### 3.2 Evaluation of structural model

The second step in evaluating PLS-SEM results involves examining the structural model [47]. According to [32, 48], and [49], the standard assessment criteria, which should be considered to assess the structural model includes five aspects, namely collinearity assessment, structural model path coefficients, coefficient of determination (R^2^), and effect size (f^2^).

#### 3.2.1 Collinearity assessment

Since the path coefficients in the structural model are based on OLS regressions, it is crucial to examine any collinearity issues to ensure they do not bias the regression results [49]. The collinearity is reported through the construct’s variance inflation factor (VIF) value. According to [49] and [51], the construct’s tolerance (VIF) value should be higher than 0.20 and lower than 5 to avoid the collinearity issue. As is evident in Table 4, the majority of the calculated VIF-variance inflation values are within the recommended threshold except the construct school environment (VIF = 5.5636). To address this issue, the problematic construct (school environment) has been eliminated before conducting the structural path analysis as directed in [32].

**Table 4:** Collinearity assessment.

Constructs	VIF	Higher than 0.20 and lower than 5
Leadership (LED)	3.405	Yes
School Environment (SE)	5.5636	No
Student’s Achievement (SA)	1.00	Yes
Teacher Quality (TQ)	4.119	Yes
Technology (TEC)	2.990	Yes

#### 3.2.2 Structural model path coefficients

Path coefficients of the structured model were assessed by employing the bootstrapping with 500 sub-samples, a two-tailed test, and a 0.05 significance level. As presented in Table 5 and Figure 1, all the hypothesized relationships are observed to be statistically significant except H_h_. H_h_ was not assessed further since the construct school environment was omitted due to a collinearity issue. However, teacher quality (β = 0.588, t = 12.242, p < 0.001), technology (β = 0.279, t = 5.402, p < 0.001) and leadership (β = 0.132, t = 2.383, p < 0.001), have positive and significant impact on student’s achievement as supporting H_b_, H_d_, and H_f_, respectively. Similarly, student’s achievement (β = 0.923, t = 92.713, p < 0.001) has a strong positive and significant impact on school quality supporting H_j_.

**Table 5:** Path coefficient.

Path Coefficient	Coefficient (Original)		Coefficient (Mean)	Std. Dev.	T Statistics	P Values
Leadership → Student’s Achievement		0.132	0.125	0.055	2.383	0.018
Student’s Achievement → School Quality		0.923	0.924	0.010	92.713	0.000
Teacher Quality → Student’s Achievement		0.588	0.592	0.048	12.242	0.000
Technology → Student’s Achievement		0.279	0.28	0.052	5.402	0.000
		R Square	R Square Adjusted			
School Quality		0.853	0.852			
Student's Achievement		0.886	0.883			

#### 3.2.3 Coefficient of determination (R^2^)

To assess a model’s explanatory power, scholars often use the coefficient of determination (R^2^) of the endogenous construct(s). R^2^ values vary from 0 to 1, with higher values suggesting stronger explanatory power. According to [32], R^2^ values of 0.75, 0.50, and 0.25 for the endogenous constructs are considered substantial, moderate, and weak, respectively. Table 5 shows that the R^2^ values for student’s achievement and school quality are 0.886 and 0.853, respectively, indicating that these two constructs have a strong R^2^ and the models have a high explanatory power [49].

#### 3.2.4 Effect size (f^2^)

Effect size often denoted as f^2^ is a statistical measure to assess the relative impact of the exogenous constructs on the endogenous constructs. According to the suggested guideline of [32] and [49], the f^2^ value of 0.02, 0.15, and 0.35 are considered as an exogenous construct’s small, medium, and large effects, respectively, on an endogenous construct. Table 6 presents that SA and TQ have large effects on SQ. However, TEC and LED have medium and small effects on SQ, respectively. In contrast, TQ, TEC, and LED have large, medium, and small effects on SA, respectively.

**Table 6:** Effect size.

	f^2^ (SQ)	Effect size	f^2^ (SA)	Effect size
Leadership (LED)	0.122	Small	0.132	Small
School Quality (SQ)	–	–	–	–
Student's Achievement (SA)	0.923	Large	–	–
Teacher Quality (TQ)	0.543	Large	0.588	Large
Technology (TEC)	0.257	Medium	0.279	Medium

#### 3.2.5 Predictive relevance (Q^2^)

To obtain cross-validated redundancy measures for each endogenous construct, this study examined predictive relevance (Q^2^) using the blindfolding procedure. The omission distance was chosen 7 as recommended in [32]. As presented in Table 7, Q^2^ values of the constructs LED, SQ, SA, and TEC are 0.623, 0.749, 0.600, 0.647, and 0.417 respectively, indicating all constructs’ predictive relevance are large.

**Table 7:** Predictive relevance.

	SSO	SSE	Q^2^	Predictive Relevance
Leadership (LED)	512	193.135	0.623	Large
School Quality (SQ)	512	128.432	0.749	Large
Student's Achievement (SA)	768	307.525	0.600	Large
Teacher Quality (TQ)	512	180.937	0.647	Large
Technology (TEC)	384	223.871	0.417	Large

## 4 Discussion

**Figure 3:**
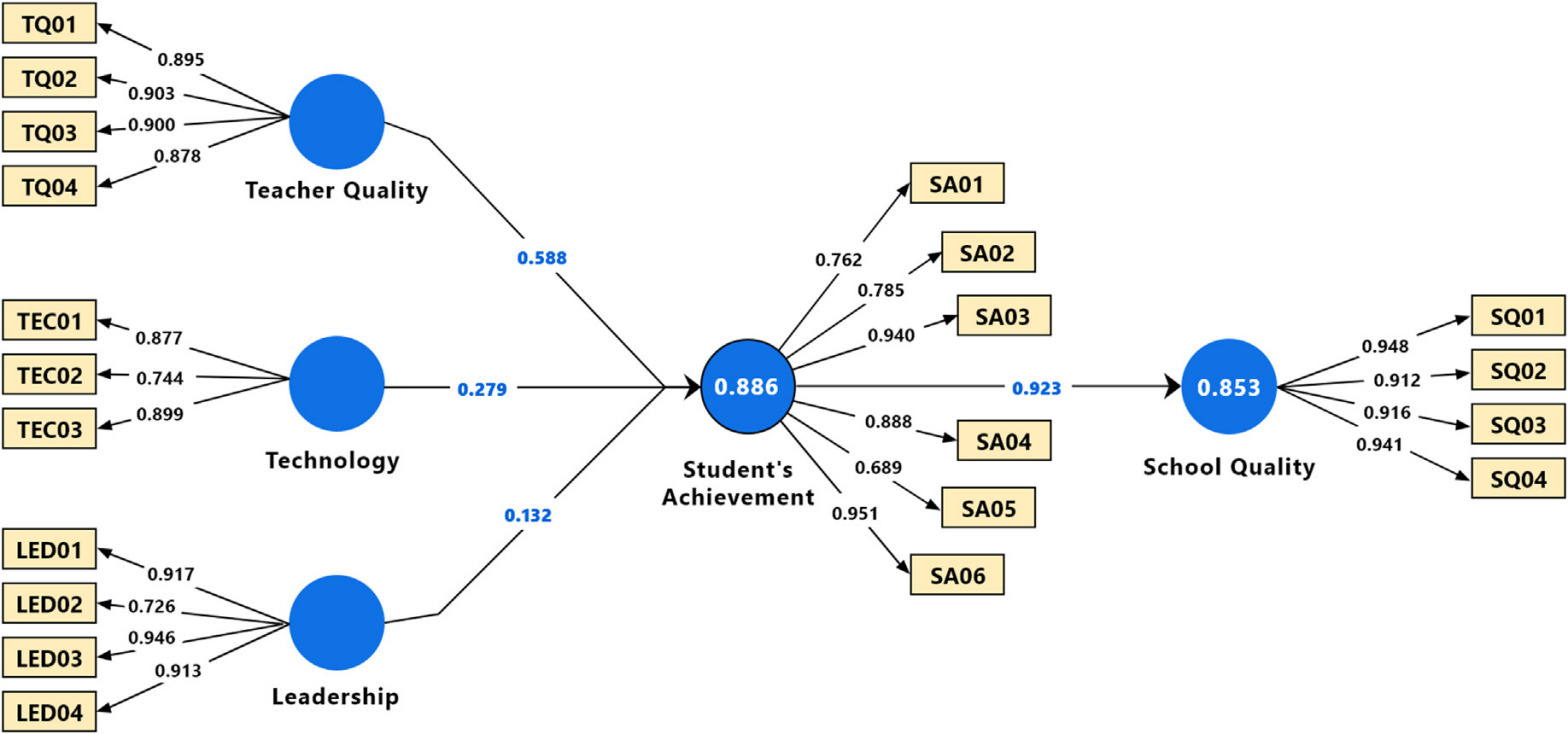
Final model.

The present study empirically revealed the impacts of constructs such as teacher’s quality, leadership, technology, school environment, and students' achievement on primary and secondary level school quality in the context of Bangladesh. Adopting partial least squares structural equation modeling (PLS-SEM), this study proposes a unique school quality assessment model (see Figure 3) in the light of previous studies. The validity and reliability of the reflective measurement model were ensured in Tables 1, 2, and 3, while the structural model was evaluated using collinearity assessment, path coefficients, model’s explanatory power, effect size, and predictive relevance in Tables 4, 5, 6 and 7, respectively.

From the structural model evaluation, it is evident that teachers' quality has a positive and significant (β = 0.588, t = 12.242, p < 0.001) impact on students’ achievement. Hence, this finding complies with N. Burroughs et al. [52], who argued that teachers play the most important role in determining students' academic achievement. The same study found that students' academic progress is enhanced if the teachers have a few years of experience, a strong academic background, and professional skills. Not only that, the findings are also compatible with prior studies [10, 53] that showed the factors related to teacher’s quality such as experience, teaching strategy, supportive behavior, and classroom management skills, etc. have significant non-linear effects on student achievement. Similar findings were obtained in studies [54, 55] and [56] where the authors revealed that qualified teachers (e.g., having subject mastery, pedagogical knowledge, self-efficacy, motivation, teaching enthusiasm, and good questioning behaviors) can significantly boost students’ interest in the subject matter and motivate them to learn, hence significantly contributing to students’ achievement.

In addition to that, this study found technology has also positive and significant (β = 0.279, t = 5.402, p < 0.001) impact on students’ achievement. The finding seems logical as technology-based learning improves students’ learning quality and ultimately improves their performance. According to a recent study [13], technology-enabled classrooms make the learning process more interesting and easier to understand, while also providing students with the opportunity to explore and learn new things. The finding is also compatible with a study [14], where the authors showed how information and communications technology (ICT) based classrooms increase students’ (primary school) learning thrust as well as achievement. While explaining the fact, the authors stated that students were more ecstatic in the classroom compared to the traditional classroom settings while utilizing Augmented Reality (AR). Another study found technology is a tool that motivates students to learn and increases their confidence level [57]. Also, technology-enhanced learning materials assist students to develop a positive attitude toward complex subjects such as mathematics through fun and practicality. As evident in a longitudinal study [58], ICT-based math skills development programs in classroom settings help students improve problem-solving and decision-making skills, as well as overcome mathematics anxiety. Moreover, according to a study [59], technology (e.g., Artificial Intelligence) improves teachers’ instructional effectiveness as well as students’ learning ability, resulting in their learning achievement.

Furthermore, the findings of this study also recommend that school leadership has a positive and significant (β = 0.132, t = 2.383, p < 0.05) effect on students' achievement. This was in line with a study [60], where the authors reveal that by taking various strategies, such as choosing passionate teachers for the school, enhancing collaboration and teacher competence, the leadership can positively influence the school environment conducive to academic success of the students. The studies [20] and [21] found that both instructional leadership and transformational leadership positively impact students' academic achievement through fixing educational goals, improving school culture and vision, planning the curriculum, and ensuring teachers' quality and their teaching. Following the Partial Least Square Path Modeling (PLS-PM) approach, Crisci et al. revealed the relationship between the affecting factors of teachers’ satisfaction: leadership, school climate, communication, involvement, structure, and job satisfaction [61]. The findings showed that leadership appears to be important in promoting teacher job satisfaction and thus interactively affects student achievement [62].

Finally, the present study found student’s achievement as a highly influential predictor (β = 0.923, t = 92.713, p < 0.001) of school quality. Hence, this finding complies with Schneider et al. [8], where the authors proposed a framework that included five categories of indicators, each classified into inputs and outputs. Among the indicators, students' academic achievement was identified as a predictor of school quality. The authors argued that parents and community members try to understand a school’s quality by using previous student achievement as a benchmark. Similar findings were also obtained in [63, 64] where the authors showed how parents choose schools for their children based on schools’ perceived academic quality.

## 5 Recommendations and policy implications

Based on the findings of the present study, the following recommendations and policy implications are suggested to enhance the overall quality of the primary and secondary level schools in the countries like Bangladesh:

i. Teachers’ professional development should be prioritized in order to enhance the teaching quality.
ii. Prior to teach, especially at primary and secondary level schools, teachers should be required to complete Standard Teachers’ Training (STT) program.
iii. To improve the learning quality in primary and secondary schools, technology-based teaching and learning facilities, as well as suitable IT infrastructure, should be implemented.
iv. Every school needs professional leadership (an individual or a group of individuals) to carry out its vision and mission.
v. An efficient quality assurance and monitoring system should be introduced in the schools for continuous monitoring and improvement.
vi. Finally, the government or the concern authorities should come up with strategic policies in order to support quality teaching-learning environment in the primary and secondary level schools.

## 6 Conclusion

Education is arguably the most important bedrock of any development. Almost all nations, including Bangladesh, consider primary and secondary education to be so crucial for individual and societal well-being that governments have made it obligatory until a certain age. However, in Bangladesh, education quality is questionable in many aspects, particularly at the primary and secondary levels. So, primary and secondary school quality indicators need to be defined, assessed, and monitored considering their incomparable contribution to the entire educational process. Aiming to explore the key “driver” constructs of school quality using PLS-SEM, this study found teacher quality, technology, and school leadership have a significant positive influence on students’ academic achievement which ultimately affects the overall school quality. Among the constructs, teachers’ quality was shown to be the most important construct that has a positive impact on students’ achievement. While students’ achievement was found to be the significant predictor of school quality. Surprisingly, though, this study found the school environment had neither effect on students’ academic achievement nor school quality. Finally, this study validates the definite link between TQ, TEC, LED, SA and SQ (see Figure 3). The overall findings should aid devolved authorities in implementing synchronized strategies to promote primary and secondary school quality.

## 7 Study limitations and research directions

Despite having many potential findings, this study acknowledges a few limitations also. Firstly, the major limitation of this study lies in the ignorance of several potential constructs, such as co-curricular activities, school culture, and the classroom, because of the limited scope of the research. Secondly, the lack of sufficient previous studies and findings in the context of Bangladesh. As a consequence, comparisons between the findings of this study along with the other studies (in the context of Bangladesh) were not furnished adequately. Finally, this study was conducted immediately after the schools reopened following the COVID-19 pandemic situation. Consequently, there might be some differences in findings from the typical time. So, in the future, additional longitudinal studies (both quantitative and qualitative) need to be done to improve the understanding and generalizability of relationships between the constructs.

## Declarations

### Author contribution statement

Suman Ahmmed: Conceived and designed the experiments; Analyzed and interpreted the data; Wrote the paper.

Jashodhan Saha: Performed the experiments; Analyzed and interpreted the data; Wrote the paper.

Maruf Ahmed Tamal: Performed the experiments; Contributed reagents, materials, analysis tools or data; Wrote the paper.

### Funding statement

This study was supported by the Institute for Advanced Research (IAR), United International University (UIU), Bangladesh [Grant Reference: UIU/IAR/01/2021/SE/03].

### Data availability statement

Data will be made available on request.

### Declaration of interest’s statement

The authors declare no conflict of interest.

### Additional information

No additional information is available for this paper.

## Appendix A Supplementary data

Questionnaire.docx Questionnaire.docx

## References

[1] SDG Resources for Educators – Quality Education. https://en.unesco.org/themes/education/sdgs/material/04.

[2] Education Statistics. https://datatopics.worldbank.org/education/wRsc/classification.

[3] Goczek Ł., Witkowska E., Witkowski B. (2021). How does education quality affect economic growth?. Sustainability.

[4] Grant C. (2017).

[5] Colclough C. (1982). The impact of primary schooling on economic development: a review of the evidence. World Dev..

[6] Arias R., Giménez G., Sánchez L. (2016). Impact of education on poverty reduction in Costa Rica: a regional and urban-rural analysis. Contemp. Rural Social Work J..

[7] Maikuva Anekeya D. (2015). School based factors affecting quality of education in primary schools in kakamega North sub county, Kenya. Int. J. Recent Res. Social Sci. Hum..

[8] Schneider J., Jacobsen R., White R., Gehlbach H. (2017). Building a better measure of school quality. Phi Delta Kappan.

[9] Mayer D.P., Mullens J.E., Moore M.T., Ralph J. (2000).

[10] Canales A., Maldonado L. (2019). Teacher quality and student achievement in Chile: linking teachers’ contribution and observable characteristics. Int. J. Educ. Dev..

[11] Harris D., Sass T. (2011). Teacher training, teacher quality and student achievement. J. Publ. Econ..

[12] Podolsky A., Kini T., Darling-Hammond L. (2019). Does teaching experience increase teacher effectiveness? A review of US research. J. Prof. Capital Commun..

[13] Phoong S., Phoong S., Moghavvemi S., Sulaiman A. (2019). Effect of Smart classroom on student achievement at higher education. J. Educ. Technol. Syst..

[14] Arvanitaki M., Zaranis N. (2020). The use of ICT in teaching geometry in primary school. Educ. Inf. Technol..

[15] Burbules N., Fan G., Repp P. (2020). Five trends of education and technology in a sustainable future. Geogr. Sustain..

[16] Taleb Z., Hassanzadeh F. (2015). Toward Smart school: a comparison between Smart school and traditional school for mathematics learning. Procedia – Social Behav. Sci..

[17] Zhang M., Li X. (2021). Design of Smart classroom system based on internet of things technology and Smart classroom. Mobile Inf. Syst..

[18] Kythreotis A., Antoniou P. (2015).

[19] Netolicky D. (2020). School leadership during a pandemic: navigating tensions. J. Prof. Capital Commun..

[20] Cruickshank V. (2017). The influence of school leadership on student outcomes. Open J. Soc. Sci..

[21] Shatzer R., Caldarella P., Hallam P., Brown B. (2013). Comparing the effects of instructional and transformational leadership on student achievement: implications for practice. Educ. Manage. Adm. Leader..

[22] Li Y., Karanxha Z. (2022). Literature Review of Transformational School Leadership: Models and Effects on Student Achievement (2006–2019). Educ. Manage. Adm. Leader..

[23] Sun J., Leithwood K. (2012). Transformational school leadership effects on student achievement. Leader. Pol. Sch..

[24] Leithwood K., Jantzi D. (2000). The effects of transformational leadership on organizational conditions and student engagement with school. J. Educ. Adm..

[25] Robinson V., Lloyd C., Rowe K. (2008). The impact of leadership on student outcomes: an analysis of the differential effects of leadership types. Educ. Adm. Q..

[26] Dutta V., Sahney S. (2016). School leadership and its impact on student achievement. Int. J. Educ. Manag..

[27] Dworkin P. (2009). Developmental-Behavioral Pediatrics.

[28] Korir D., Kipkemboi F. (2014). The impact of school environment and peer influences on students’ academic performance in Vihiga county, Kenya. J. Educ. Pract..

[29] Lombardi E., Traficante D., Bettoni R., Offredi I., Giorgetti M., Vernice M. (2019). The impact of school climate on well-being experience and school engagement: a study with high-school students. Front. Psychol..

[30] Bangladesh Bureau of Educational Information and Statistics" (2022). Banbeis.portal.gov.bd. http://banbeis.portal.gov.bd/.

[31] do Valle P., Assaker G. (2015). Using partial least squares structural equation modeling in tourism research. J. Trav. Res..

[32] Hair J., Hult G., Ringle C., Sarstedt M. (2016).

[33] Ferine K., Aditia R., Rahmadana M., Indri (2021). An empirical study of leadership, organizational culture, conflict, and work ethic in determining work performance in Indonesia’s education authority. Heliyon.

[34] Kock N., Hadaya P. (2016). Minimum sample size estimation in PLS-SEM: the inverse square root and gamma-exponential methods. Inf. Syst. J..

[35] Kock N., Ali F., Rasoolimanesh S.M., Cobanoglu C. (2018). Applying Partial Least Squares in Tourism and Hospitality Research.

[36] Faul F., Erdfelder E., Buchner A., Lang A.-G. (2009). Statistical power analyses using G∗ power 3.1: tests for correlation and regression analyses. Behav. Res. Methods.

[37] Ashraf M., Ahmed H. (2022). Approaches to quality education in tertiary sector: an empirical study using PLS-SEM. Educ. Res. Int..

[38] Sukendro S. (2020). Using an extended Technology Acceptance Model to understand students’ use of e-learning during Covid-19: Indonesian sport science education context. Heliyon.

[39] Haynes S., Richard D., Kubany E. (1995). Content validity in psychological assessment: a functional approach to concepts and methods. Psychol. Assess..

[40] Boateng G., Neilands T., Frongillo E., Melgar-Quiñonez H., Young S. (2018). Best practices for developing and validating scales for health, social, and behavioral research: a primer. Front. Public Health.

[41] McHugh M. (2012). Interrater reliability: the kappa statistic. Biochem. Med..

[42] Kawakami N. (2020). Internal consistency reliability, construct validity, and item response characteristics of the Kessler 6 scale among hospital nurses in Vietnam. PLoS One.

[43] Tavakol M., Dennick R. (2011). Making sense of Cronbach’s alpha. Int. J. Med. Educ..

[44] Ringle C., Wende S., Becker J. (2015). SmartPLS 3.3.9. SmartPLS.

[45] Dash G., Paul J. (2021). CB-SEM vs PLS-SEM methods for research in social sciences and technology forecasting. Technol. Forecast. Soc. Change.

[46] Choosing Between Pls-SEM and CB-SEM – SmartPLS. https://www.smartpls.com/documentation/choosing-pls-sem/choosing-between-pls-sem-and-cb-sem/.

[47] Fornell C., Larcker D. (1981). Evaluating structural equation models with unobservable variables and measurement error. J. Market. Res..

[48] Hair J., Risher J., Sarstedt M., Ringle C. (2019). When to use and how to report the results of PLS-SEM. Eur. Bus. Rev..

[49] Hair J., Hult G., Ringle C., Sarstedt M., Danks N., Ray S. (2021).

[50] Sarawa D., Mas'ud A. (2020). Strategic public procurement regulatory compliance model with mediating effect of ethical behavior. Heliyon.

[51] Becker J., Ringle C., Sarstedt M., Völckner F. (2015). How collinearity affects mixture regression results. Market. Lett..

[52] Burroughs N. (2019).

[53] Lee S., Lee E. (2020). Teacher qualification matters: the association between cumulative teacher qualification and students’ educational attainment. Int. J. Educ. Dev..

[54] Fauth B. (2019). The effects of teacher competence on student outcomes in elementary science education: the mediating role of teaching quality. Teach. Teach. Educ..

[55] Olagbaju O. (2020). Teacher-related factors as predictors of students’ achievement in English grammar in Gambian senior secondary schools. Educ. Res. Int..

[56] Keller M., Neumann K., Fischer H. (2016). The impact of physics teachers’ pedagogical content knowledge and motivation on students’ achievement and interest. J. Res. Sci. Teach..

[57] Raposo A., Durão A., Estradas A., Ribeiro I. (2020). Technology as a tool to enhance motivation and learning. E3S Web Conf..

[58] Saha J., Ahmmed S., Ali M., Tamal M., Rezaul K. (2020). ICT based mathematics skill development program: an initiative to overcome mathematics anxiety. Int. J. Emerg. Technol. Learn. (iJET).

[59] Srinivasan V., Murthy H. (2021). Improving reading and comprehension in K-12: evidence from a large-scale AI technology intervention in India. Comput. Educ.: Artif. Intell..

[60] Huguet B. (2017). Effective leadership can positively impact school performance. Horizon.

[61] Crisci A., Sepe E., Malafronte P. (2018). What influences teachers’ job satisfaction and how to improve, develop and reorganize the school activities associated with them. Qual. Quant..

[62] Banerjee N., Stearns E., Moller S., Mickelson R. (2017). Teacher job satisfaction and student achievement: the roles of teacher professional community and teacher collaboration in schools. Am. J. Educ..

[63] Hofflinger A., Gelber D., Tellez Cañas S. (2020). School choice and parents’ preferences for school attributes in Chile. Econ. Educ. Rev..

[64] Bast J., Walberg H. (2004). Can parents choose the best schools for their children?. Econ. Educ. Rev..

