# Supplementary material for: An empirical study for determining the quality indicators for the primary and secondary school of Bangladesh: A structural equation modeling approach
Source: Heliyon. 2022 Oct 3;8(10):e10870. doi: 10.1016/j.heliyon.2022.e10870 (PMC9562340; doi:10.1016/j.heliyon.2022.e10870)
Supplement: Questionnaire.docx [file mmc1.docx]

**Appendix A.**

| **Factors Affecting Primary and Secondary Level School Quality** |
| --- |
| The following questions should be read carefully by appraiser. Please be truthful in your answers. This data will only be used for research purposes. The schools identify will not be revealed. Use tick [√] mark for giving your valuable answer. |

| **S/N** | **Indicators** | **Questions** | **Options** |
| --- | --- | --- | --- |
| **1** | **B01** | Institution’s Name | ________________________ |
| **2** | **B02** | Institution’s Location | Rural/Sub-urban/Urban |
| **3** | **B03** | Institution’s Level | Primary/High-school/College |
| **4** | **B04** | District | ________________________ |
| **5** | **TQ01** | The teachers of this institution have strong academic background/skills. | Strongly disagree/ Disagree/ Neutral/ Agree/ Strongly Agree |
| **6** | **TQ02** | The teachers of this institution have sufficient experience in their field of teaching. | Strongly disagree/ Disagree/ Neutral/ Agree/ Strongly Agree |
| **7** | **TQ03** | The teachers of this institution are professional enough (in teaching). | Strongly disagree/ Disagree/ Neutral/ Agree/ Strongly Agree |
| **8** | **TQ04** | The teachers of this institution have training in the field of their teaching. | Strongly disagree/ Disagree/ Neutral/ Agree/ Strongly Agree |
| **9** | **TEC01** | Students and teachers of this institution are familiar technology-based teaching-learning. | Strongly disagree/ Disagree/ Neutral/ Agree/ Strongly Agree |
| **10** | **TEC02** | This institution has digital/multimedia-based classroom(s). | Strongly disagree/ Disagree/ Neutral/ Agree/ Strongly Agree |
| **11** | **TEC03** | The staffs (academic & administrative) of this institution have sound IT knowledge. | Strongly disagree/ Disagree/ Neutral/ Agree/ Strongly Agree |
| **12** | **LED01** | This institution has specific goals, mission and vision. | Strongly disagree/ Disagree/ Neutral/ Agree/ Strongly Agree |
| **13** | **LED02** | This institution has individual or group of individuals who continuously provide direction, guidance, and support in the school’s journey toward achieving its goals. | Yes/ No |
| **14** | **LED03** | The head master/principal of this intuition has strategic thinking and innovation mindset for the betterment of this institution. | Strongly disagree/ Disagree/ Neutral/ Agree/ Strongly Agree |
| **15** | **LED04** | Teachers and staffs have confidence in the institution’s leadership. | Strongly disagree/ Disagree/ Neutral/ Agree/ Strongly Agree |
| **16** | **SE01** | Please rate the overall indoor environment (e.g., classroom, library, teachers' room, principal's room, working environment etc.). | 1/2/3/4/5/6/7/8/9/10 |
| **17** | **SE02** | Please rate the overall outdoor environment (e.g., sufficient space, green campus, cleanliness) (rate out of 10). | 1/2/3/4/5/6/7/8/9/10 |
| **18** | **SE03** | Overall teaching-learning environment of this institution is satisfactory. | Strongly disagree/ Disagree/ Neutral/ Agree/ Strongly Agree |
| **19** | **SA01** | GPA-5 (A+) achievement (last 5 year average). | ____________________ |
| **20** | **SA02** | Students’ passing rate (last 5 year average). | ____________________ |
| **21** | **SA03** | The board results of the students in this institution (rate out of 10). | 1/2/3/4/5/6/7/8/9/10 |
| **22** | **SA04** | Every year a significant number of students of this institution get scholarships/ get chance in reputed government university. | Strongly disagree/ Disagree/ Neutral/ Agree/ Strongly Agree |
| **23** | **SA05** | Every year a significant number of students of this institution get awards in co-curricular activities. | Strongly disagree/ Disagree/ Neutral/ Agree/ Strongly Agree |
| **24** | **SA06** | The overall performance of the students in this institution is (rate out of 10): | 1/2/3/4/5/6/7/8/9/10 |
| **25** | **SQ01** | This institution has a great reputation due to its good teaching-learning quality. | Strongly disagree/ Disagree/ Neutral/ Agree/ Strongly Agree |
| **26** | **SQ02** | The enrollment of this institution is growing every year due to its good reputation. | Strongly disagree/ Disagree/ Neutral/ Agree/ Strongly Agree |
| **27** | **SQ03** | Every year a significant number of students of this school achieve outstanding results in board exam. | Strongly disagree/ Disagree/ Neutral/ Agree/ Strongly Agree |
| **28** | **SQ04** | Parents are willing and worry-free about sending their children to this school. | Strongly disagree/ Disagree/ Neutral/ Agree/ Strongly Agree |
